# Molecular evidence for a new endemic species of *Acartia* (Copepoda, Calanoida) from the Southeast Pacific coast

**DOI:** 10.1038/s41598-024-62080-5

**Published:** 2024-05-29

**Authors:** Andrés Mesas, Víctor M. Aguilera, Carolina E. González, Ricardo Giesecke, Rubén Escribano, Cristian A. Vargas

**Affiliations:** 1 Millennium Institute of Oceanography, Universidad de Concepción, Concepción, Chile; 2 Coastal Ecosystems and Global Environmental Change Lab (ECCALab), Department of Aquatic System, Faculty of Environmental Sciences, Universidad de Concepción, Concepción, Chile; 3 Centro de Estudios Avanzados en Zonas Áridas (CEAZA), Bernardo Ossandón #877, C.P. 1781681 Coquimbo, Chile; 4 Departamento de Biología Marina, Facultad de Ciencias del Mar, Universidad Católica del Norte, Coquimbo, Chile; 5 Instituto de Ciencias Marinas y Limnológicas, Universidad Austral de Chile, Valdivia, Chile; 6 Department of Oceanography, Faculty of Natural and Oceanographic Sciences, University of Concepción, 4030000 Concepción, Chile; 7 Centro de Investigación Dinámica de Ecosistemas Marinos de Altas Latitudes (IDEAL), Universidad Austral de Chile, Valdivia, Chile; 8 Coastal Social-Ecological Millennium Institute (SECOS), Universidad de Concepción & P. Universidad Católica de Chile, Santiago, Chile

**Keywords:** Phylogeny, COI, 18 s, Population genetics, New endemic species, *Acartia tonsa*, Cryptic species, Copepods, Southeast Pacific Ocean, Ecology, Biodiversity, Population genetics, Marine biology

## Abstract

The loss of biodiversity in marine populations is one of the consequences of the increased events of extreme environmental conditions in the oceans, which can condition the persistence of populations to future scenarios of climate change. Therefore, it is extremely necessary to explore and monitor the genetic diversity of natural populations. In the Southeast Pacific Ocean (SEPO), specifically on the coast of Chile, the presence of the copepod *Acartia tonsa* has been indicated solely using morphological evidence, due to the absence of genetic information. In the present work, the genetic diversity, population structure and phylogenetic position within the genus *Acartia*, of populations identified morphologically as *A. tonsa*, was evaluated by amplification of the mitochondrial cytochrome c oxidase subunit I and nuclear marker 18 s. Our results showed that the populations identified as *A. tonsa* correspond to a new monophyletic group endemic to SEPO (GMYC = 1.00; PTP = 0.95). The populations showed moderate to high genetic diversity with an incipient structuring between populations and biogeographic zones. Our results suggest that despite the homogenizing effect of the Humboldt Current, isolation by distance and contrasting environmental conditions at different geographic scales have an important influence on the genetic diversity of zooplankton in the SEPO region.

## Introduction

A comprehensive understanding of biological diversity, including its individual components, has become a paramount objective within the scientific community studying marine ecosystems. This heightened emphasis from the escalating environmental alterations induced by climate change and exacerbated human activities during the Anthropocene, are leading to a deterioration of biodiversity^1–3^. Given that oceans serve as primary sinks for greenhouse gasses and atmospheric heat, resulting in ongoing ocean acidification and warming^4,5^, it is reasonable to anticipate that the primary indicators of biodiversity loss will become evident in marine ecosystems^6–8^.

The species distribution and the genetic diversity that populations harbor can serve as indicators of the natural population’s capacity to adapt to environmental variations and extreme conditions in their habitats^9,10^. Current changes in oceanic conditions have influenced the distribution of numerous marine species, with many either shifting or expanding their ranges searching suitable environments for development^11,12^. In practical terms, distributional changes can either enhance or restrict the connectivity between populations, potentially reshaping the current and future diversity of natural populations^13–15^. In this context, a recurrent assessment of genetic diversity within natural populations is needed, with a specific focus on endemic and cosmopolitan species.

In marine environments, owing to lower apparent barriers to dispersal, species with high dispersal potential are more likely to display reduced genetic diversity and population structuring, possibly even less than what is observed in terrestrial ecosystems^16–18^. Nevertheless, it has been observed that species with varying degrees of vagility, including copepods^19,20^, nudibranchs^21^, shrimps^22^, and fishes^23^, exhibit substantial genetic diversity and population structure, often with molecular divergence reaching interspecific levels. Molecular techniques have revealed instances in which species previously believed to have a broad distribution actually consist of multiple species that cannot be distinguished morphologically and harbor a completely unknown cryptic diversity^20,24,25^.

While it reaffirms the fact that diversity in the marine environment is still underestimated, it also poses a problem because accurate estimations of each component of biodiversity begins with biological classification and species identification. Many of the cosmopolitan species inhabiting the Pacific Ocean were initially described based on populations from the Atlantic Ocean, Indian Ocean, or the Mediterranean Sea, due to the late discovery and description of the biota of the Pacific Ocean and the accidental transport of many species during the maritime trade voyages of the Silk Road (West Pacific coast) and trade with European colonies in the New World (East Pacific coast; see details in^26^). Consequently, the Pacific Ocean provides an ideal scenario for studying cryptic biodiversity and population divergence.

The calanoid copepod *Acartia tonsa* Dana, 1849, is classified as a cosmopolitan species, distributed along the coasts of the southeastern Pacific Ocean (SEPO) and commonly inhabiting estuaries and coastlines along the Indian Ocean, Pacific Ocean, Atlantic Ocean, as well as the Mediterranean and Baltic Seas^27,28^. The *Acartia* ability to tolerate a broad range of salinity (0.3–36.9 psu) and temperature (− 1 to 32 °C)^29,30^ enables this species to achieve a wide distribution. Consequently, its ecology and evolution has been extensively studied^31–34^. At the central and southern coast of SEPO, the population density of *A. tonsa* depends mainly on food availability (mainly microplankton), exhibiting a marked seasonality in abundance on the central and southernmost coast of SEPO^35,36^. *A. tonsa* is usually the dominant copepod species in embayment’s, estuaries, fjords, sounds, and inner channel systems, acting as an important link between primary producers and higher trophic levels^37^.

Phylogeographic and phylogenetic studies conducted on *A. tonsa* along the western coast of the Atlantic Ocean have revealed that populations from both the northern and southern hemispheres exhibit profound molecular divergences, suggesting the presence of a complex of cryptic species in sympatry that have developed prezygotic mechanisms of reproductive isolation^38–44^. The broad thermal and salinity tolerances exhibited by several *A. tonsa* lineages may contribute to differences in their habitat utilization patterns, resulting in niche partitioning^39^. On a larger geographic scale, the genetic diversity of *A. tonsa* is strongly influenced by ocean currents and coastal geography^44^. However, the complexity of recognizing diagnostic characters among congeneric species of the genus *Acartia* has led to confusion in delineating lineages within *A. tonsa* populations along the Brazilian coast^41,42,45^. To date, the presence of *A. tonsa* in SEPO coastal systems has been identified solely based on morphological characteristics, without molecular confirmation. In this context, it is crucial to emphasize that despite multiple studies on the distribution, physiology, and life history of populations along the coasts of Chile, there are no molecular sequences of *A. tonsa* available for the SEPO^34,46–49^. The SEPO, specifically the coastal domain along the Chilean coast (18–56° S), provides a unique natural setting for studying the population dynamics of planktonic coastal species. The Humboldt Current System (18–42° S), in combination with the coastal upwelling (from the equator to 40° S), the increasing complexity of the coastal geography towards the southernmost localities (fjords and canals of Patagonia) and the contribution of fresh water from rivers and glaciers, which is highest in the southernmost places (south of 40° S), creates favorable scenarios for the emergence of ruptures of population connectivity and local adaptation to specific environmental conditions^50–52^. Many coastal species exhibit phylogenetic breaks along the SEPO associated with biogeographic discontinuities, especially those reported between 30 and 32° S (*Acanthina monodon, Scurria scurra, Tegula atra,* and *Orchestoidea tuberculata* in^16^; *Pyura chilensis* in^53^), which coincides with the southern limit of the Peruvian province^54^. On the other hand, around 42° S, a divergence occurs in the South Pacific Current, leading to the formation of the Humboldt Current to the north and the Cape Horn Current to the south. This natural barrier contributes to the isolation of marine fauna^55^, while other marine species with higher dispersal capacity show no population structure at a macrogeographic scale (*Concholepas concholepas* in^56^; *Pleuroncondes monodon, Emerita analoga, and Petrolisthes violaceus* in^16^). Therefore, it is intriguing to investigate whether various discontinuities in oceanographic conditions, geographical distances, and historical events, such as the last maximum glacial period^57,58^, have played a role in shaping the genetic diversity of this widely distributed copepod species in this ocean region.

**Figure 1:**
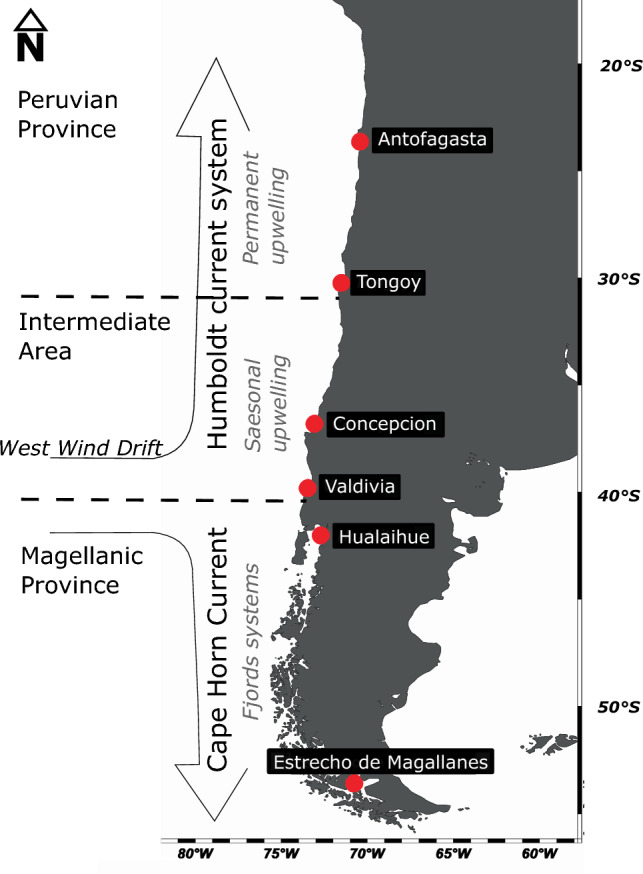
Localities studied along the coast of Chile (SEPO 23–56° S), which are influenced by the Humboldt current system and the Cape Horn current. In this area there are three biogeographic areas, the Peruvian Province where there is a permanent upwelling, the Intermediate Area (transition zone) where the influence of upwelling is seasonal and the Magellanic Province where the influence of fresh water from the fjords predominates.

In this study, we investigated the genetic diversity, population structure, and phylogenetic position of morphologically identified populations of *A. tonsa* in six locations spanning over 3000 km, representing three biogeographic zones: Peruvian Province, Intermediate Area, and Magellanic Province^54^. These zones are strongly characterized by distinct water masses separated by large current systems, the presence of coastal upwelling, diverse seasonality, and freshwater input from rivers and glaciers (Fig. 1). Our study, for the first time, analyzed genetic sequences of populations identified as *A. tonsa* along the SEPO, revealing the existence of a previously undiscovered cryptic species within the genus *Acartia*.

## Results

### mtCOI marker

**Figure 2:**
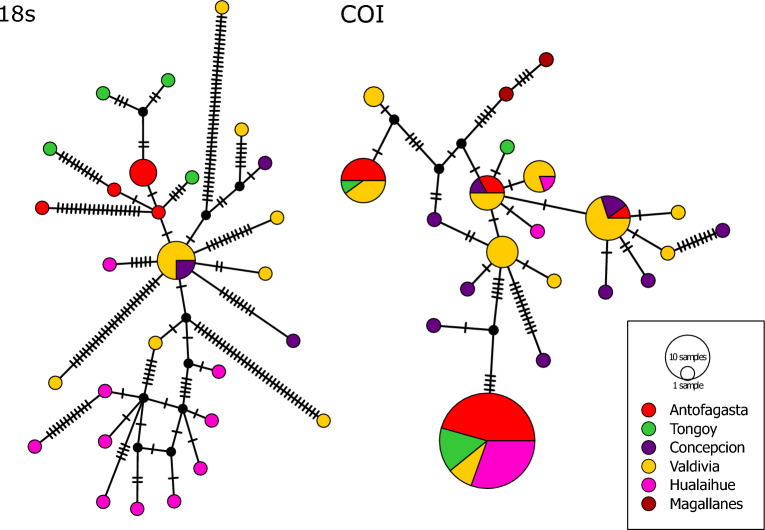
Haplotype networks constructed with minimum spanning criteria for COI and 18 s sequences obtained from the studied populations. Haplotypes are represented with a circle; whose size is proportional to its frequency and colors represent its presence in each locality studied. Lines between haplotypes represent mutational steps and black dots represent possible not sampled haplotypes.

A total of 98 mtCOI sequences were obtained from the studied locations (GenBank Accession Number: OR759115–OR759134), which were manually reviewed and edited. These sequences were aligned, resulting in an alignment of 445 base pairs (bp) in length with 54 polymorphic sites. Overall, the populations exhibited moderate nucleotide diversity (0.017 ± 0.001) and haplotypic diversity (0.756 ± 0.042), with a total of 21 haplotypes. In particular, a high diversity was found in the Intermediate area, localities of Concepcion and Valdivia, with the presence of 10 haplotypes in each one, of which 7 and 5 were private haplotypes (Table 1, Fig. 2). The most common haplotype was present in the three biogeographic zones studied, except for the populations of Concepcion and Magallanes (see details in Fig. 2). Regarding to geographical patterns in the haplotype network, no evident pattern of phylogeographic structuring was observed visually, and the analysis of AMOVA did not identify genetic differences between the biogeographic zones (Фct: 0.289, *p* value: 0.115), primarily due to a significative genetic variability within (Фst: 0.476, *p* value: 0.001) and among populations (Фsc: 0.264, *p* value: 0.003), with 52 and 19% of genetic variation, respectively (Table 2). The pairwise analysis of Фst showed that the populations of the Intermediate area (Concepcion and Valdivia) did not differ from each other, but both exhibited significant differences from the populations present in both biogeographic provinces (Suppl. Table S1). Interestingly, the populations of Antofagasta and Tongoy, present in Peruvian province, do not show significant differences with those of Hualaihue; located 1300 km south at the Magellan province, while Magellan population shows a high differentiation respect to other populations (Suppl. Table S1). Despite this, linearized genetic differentiation according to the geographical distance between populations, showed a positive and significant linear relationship (see details in Suppl. Fig. 1).

**Table 1:** Genetic diversity indices for the COI mitochondrial and 18 s nuclear marker for studied populations.

Locality	*n*	*na*	*np*	*h* ± *SD*	*π* ± *SD*	*p*	*nm*	*k*
*COI*								
Antofagasta	29	4	0	0.456 ± 0.101	0.012 ± 0.0026	17	17	5.433
Tongoy	9	3	1	0.417 ± 0.191	0.012 ± 0.0052	17	17	5.11
Concepcion	10	10	7	0.978 ± 0.054	0.017 ± 0.0048	34	36	7.68
Valdivia	32	10	3	0.893 ± 0.025	0.013 ± 0.0019	21	22	5.842
Hualaihue	16	3	1	0.24 ± 0.135	0.0064 ± 0.0035	13	13	2.816
Magallanes	2	2	2	1 ± 0.5	0.0091 ± 0.0046	4	4	4
All	98	21	–	0.756 ± 0.042	0.017 ± 0.001	54	57	7.471
*18 s*								
Antofagasta	7	4	4	0.86 ± 0.14	0.0084 ± 0.005	43	43	12.7
Tongoy	4	4	4	1 ± 0.18	0.018 ± 0.005	51	53	26.8
Concepcion	4	4	2	1 ± 0.17	0.006 ± 0.0012	18	18	9
Valdivia	13	8	7	0.81 ± 0.11	0.015 ± 0.005	124	133	5.8
Hualaihue	10	10	10	1 ± 0.045	0.0099 ± 0.0023	49	53	14.7
All	38	29	–	0.95 ± 0.026	0.011 ± 0.0022	161	177	14.68

*n* number of samples, *na* number of haplotypes, *np* number of private haplotypes, *h* haplotype diversity (± SD, standard deviation), *π* nucleotide diversity (± SD, standard deviation), *p* number of polymorphic sites, *nm* number of mutations, *k* the mean of the nucleotide differences.

**Table 2:** Analysis of Molecular variance (AMOVA) from COI and 18 s sequences for the studied populations.

Variation	df	Ss	sigma^2^	%variation	Fixation indices		*p *value
*COI*							
Among biogeographic zones	2	1.37	15	28.87	Фct:	0.289	0.115
Among populations	3	3.92	10	19	Фsc:	**0.264**	**0.003**
Within populations	92	2.45	27	52.3	Фst	**0.476**	**0.001**
Total	97	4.22	51				
*18 s*							
Among biogeographic zones	2	1.67	63	25	Фct:	**0.291**	**0.001**
Among populations	2	203	− 11	− 5	Фsc:	− 0.07	0.706
Within populations	33	5.38	163	75	Фst	**0.240**	**0.001**
Total	37	7.25	215				

Significant values are in bold.

### Nuclear marker 18 s

For the nuclear marker 18 s, 38 sequences were obtained (GenBank Accession Number: OR771437–OR771462), which were used to create an alignment of 1736 bp, and 161 polymorphic sites. Sequences of the 18 s marker could not be obtained from Magallanes individuals due to the high degradation of their DNA. The populations exhibited a moderate nucleotide diversity (0.011 ± 0.002) and a high haplotype diversity (0.952 ± 0.026), with 28 haplotypes (Table 1). In particular, the Hualaihue population from Magellanic Province, showed a higher number of haplotypes (10), all of which were private haplotypes (see more details in Table 1). The most common haplotype was found only in localities from the Intermediate area, populations of Concepcion and Valdivia, indicating a high presence of unique haplotypes for this nuclear marker in the studied populations (Fig. 2). Regarding the topology of the haplotype network, it was observed that the most common haplotype was centrally located, similar to the star-like pattern, which gives rise to the remaining haplotypes, entirely private. The AMOVA analysis revealed a significant variation within populations, with a 75% of genetic variation (Фst: 0.24, *p* value: 0.001), and a significative differentiation among biogeographic zones with a 25% of variation (Фct: 0.291, *p* value: 0.001, see details in Table 2). The pairwise analysis of Фst showed that the main differences were due to the fact that the population of Hualaihue, located in the Magellanic province, differed significantly from the rest of populations; similarity, the populations of Intermediate area, Concepcion and Valdivia did not differ from each other, but showed significant differences with respect to populations present in both biogeographic provinces (see in Suppl. Table S1). Linear regression with linearized genetic differentiation with respect to geographical distance between populations, also showed a positive and significant linear relationship (Suppl. Fig. S1).

#### Phylogenetic position and species delimitation

**Figure 3:**
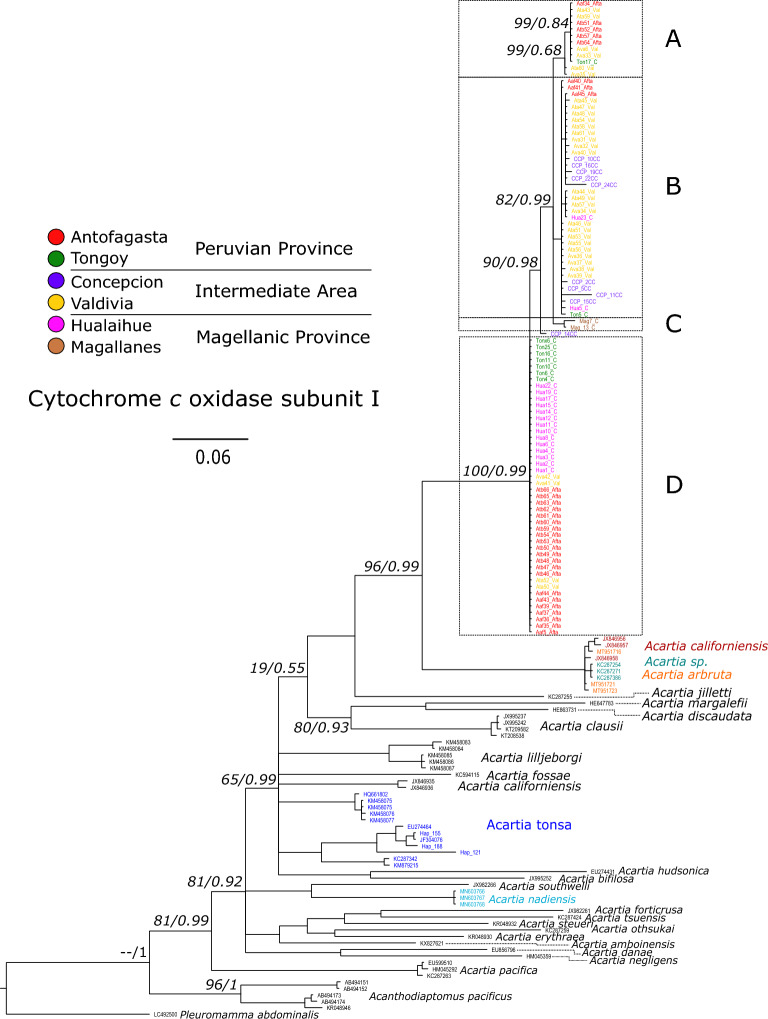
Bayesian phylogeny performed with 157 COI sequences of species of genus *Acartia sp.*, including 98 sequences obtained from the populations under study (Antofasta, Tongoy, Concepcion, Valdivia, Hualaihue and Magallanes). Maximum likelihood/Bayesian posterior probability support values were labeled in each node. Putative lineages among populations from Chilean coasts were identified with A-D letters.

Phylogenetic reconstructions by ML and BI were performed with 98 COI sequences previously obtained, plus 59 sequences (157 sequences in total) from species of the genus *Acartia* available online from GenBank (National Center for Biotechnology Information). Both inferences produced similar tree topologies between themselves and both coincided in recovering the presence of a monophyletic clade, with high bootstrap values (MLb) and posterior probability (Bpp), which grouped all the individuals of the populations studied (Fig. 3). Inside, there are four clades (A–D) that are highly related, where clade A contains individuals of Antofagasta, Tongoy, and Valdivia, the clade B mainly includes individuals of Intermediate area, Concepcion and Valdivia plus some individuals from the other biogeographical provinces. The clade C included only individuals of Magellan, and the clade D, with the largest number of individuals, brings together Hualaihue individuals as well as individuals from Antofagasta, Tongoy, and Valdivia (Fig. 3). The genetic divergence (gd) between these clades, calculated as the sum of the branch lengths of the Bayesian tree, were small and ranged from 0.0147 between the B-C clades to 0.0899 between the A-D clades (Table 3).

**Table 3:** Genetic divergences calculated as the sum of the branch lengths to mitochondrial lineages (A–D) present along SEPO coast and related Acartia species.

	Lineages within the studied populations			
	A	B	C	D
A	-	-	-	-
B	0.02	-	-	-
C	0.0167	0.0147	-	-
D	0.0899	0.0447	0.0219	-
More related taxa				
Acartia sp.	0.2317			
Acartia jilletti	0.3107			
Acartia clausii	0.3657			
Acartia margalefi	0.4495			
Acartia discaudata	0.4134			
Acartia tonsa	0.3343			

Significant values are in bold.

The phylogenetic reconstruction revealed with high support (MLb and Bpp) that the studied papulations are a sister clade (gd = 0.232) of a set of species, highly related between them, designated as *Acartia arbruta, Acartia californiensis* and *Acartia *sp., all collected from the North Pacific and whose specific identity is subject to discussion (see below). Among the following more closely related *Acartia* species are *A. jilletti* (gd = 0.311), *A. clausii* (gd = 0.366), *A. margalefi* (gd = 0.449), *A. discaudata* (gd = 0.413) and *A. tonsa* (gd = 0.334; more details in Table 3 and Fig. 3). Additionally, a phylogenetic reconstruction was performed by ML and BI with 58 sequences of the 18 s nuclear marker, 38 sequences from the populations studied, and 20 sequences from species of the genus *Acartia* available online from GenBank (National Center for Biotechnology Information). The phylogenetic reconstructions showed the studied populations as a monophyletic clade and sister to *Acartia tonsa*, with *A. hongi*, *A. omorii* and *A. clausii* being the next most closely related species (Suppl. Fig. S2). Due to the limited availability of 18S nuclear marker sequences for species of the genus *Acartia* and the fact that mitochondrial markers are mainly used in the literature, the analysis of interspecific genetic divergence and species delimitation will be performed using only the COI mitochondrial marker.

In terms of species delimitation, the two analytical approaches applied, Generalized Mixed Yule Coalecent (GMYC) and Poisson Tree Process (PTP) with 157 COI sequences used in phylogenetic reconstruction support with a high probability that the differentiation of the monophyletic clade with the populations of the coasts of Chile reaches the species level (GMYC = 1.00, PTP = 0.95) with respect to other species included in phylogeny.

## Discussion

The recognition of diagnostic traits is the first step in species identification and it is often the only one, because it is assumed that diversification is accompanied by an evident phenotypic change. The lack of capacity to differentiate morphologically similar species is a problem that has become more recurrent and relevant in taxonomy and manifested in the present study, because cryptic species are more common than expected^59,60^.

Our research showed that along the Chilean coast (SEPO region between 23° and 53° S), the use of key traits for the identification of the copepod *A. tonsa* identified individuals who formed a monophyletic group distinct from any species of the genus *Acartia*, which had not been previously reported and which seems to be endemic to the coasts of the Southeastern Pacific Ocean (SEPO). This is not the first time that the morphological traits of *A. tonsa* cryptically harbor the presence of other species, either previously described^41,42,45^ or not previously discovered^61^, as is well known for the cryptic species complex within *A. tonsa* in the NW Atlantic Ocean^39,40^. Recently, a study incorporating genetic data, morphology and phenology, identified the presence of a new cryptic species, designated as *Acartia arbruta*, which had previously been morphologically confused with *A. tonsa* in the Northeast Pacific Ocean^61^. This new species, both in the work in which it was described^61^ and in our phylogenetic analyses, was positioned in a clade in which the specific identity of its members was not well defined and indicated as *Acartia* sp. (see details in^44^). This background is relevant to understand the diversification of the genus *Acartia* in the Pacific Ocean, because the clade of *A. arbruta* was composed entirely of OTU (Operative Taxonomic Unities) collected from the Northeast Pacific Ocean, which was the sister clade of the populations included in our study, which so far are the only representatives of the SEPO. The genus *Acartia* is comprised of 65 recognized species^62,63^, of which only a dozen species belonging to the subgenera Acanthacartia, Acartia, and Odontacartia are present in the Pacific Ocean. However, recent discoveries of *A. arbruta*^61^, *A. (Odontacartia) edentata*^63^, *Acartia (O.) nadiensis*^64^, and the new monophyletic group reported by us in this study showed that the diversity of the genus *Acartia* in the Pacific Ocean is still underestimated, mainly due to confusion with species previously described in other oceans. On the other hand, our results and the presence of new endemic species in the Pacific Ocean for the genus *Acartia*^61,63,64^ suggest a wide diversification of the genus along the SEPO that needs to be studied.

In our phylogenetic analysis, the populations studied here together with the species *A. arbruta* and *A. jilletti* formed a monophyletic clade of endemic species to the Pacific Ocean, whereas species *A. nadiensis*, described from Fiji coast, was most closely related to *A. southwelli*, a species endemic to the Indian Ocean, and other *Acartia* species with a broader distribution (Fig. 3). Therefore, in view of this limited evidence, we could propose that the arrival of the genus *Acartia* to the Pacific Ocean has occurred on at least two occasions: an older one, from which diversified the species that inhabit the Indo-Pacific and Northwest Pacific Ocean, and another more recent event, from which diversified species inhabiting New Zealand coasts, Northeast and Southeast Pacific Ocean. It is necessary to consider that the experimental design of our research was not developed to test the diversification of the genus *Acartia*, so it should be appropriately studied.

Phylogenetic reconstructions using the COI marker revealed the presence of four closely related mitochondrial lineages (A–D, Fig. 3) within the studied populations, which were distributed across biogeographic areas, but were not present in all populations studied. In this sense, in all biogeographic areas, three of the four mitochondrial lineages were found, as well as in the populations of Antofagasta, Tongoy, and Valdivia we found that three of the four mitochondrial lineages were in sympatry. In contrast, in the populations of Concepcion and Magallanes, only one mitochondrial lineage was found. It is possible that the presence of a single endemic lineage in Magallanes population was produced by isolation by distance from the other populations studied, but the case of the population of Concepcion was different, because it is located between populations that showed three of the four lineages. In addition, lineage D, which was the most abundant and was present from Hualaihue to northern Chile (Antofagasta), but was not present in Concepcion, which could be due to special environmental conditions in the area. Although our study represents the first assessment of genetic diversity within the Concepcion and Magallanes populations, more sampling and research efforts are needed to confirm the existence of a single mitochondrial lineage. In large and panmictic populations, a small number of haplotypes are expected to dominate in frequency and the vast majority of genetic diversity is expected to be captured with small sample sizes, as observed in the haplotype accumulation curves. However, the use of DNA barcoding has shown that the genetic diversity of marine populations is underestimated, especially in populations that are difficult to identify and sample^65–67^. Although increasing the sampling effort is not the definitive solution, because ecological characteristics of the species studied, such as the reproductive strategy, mitochondrial inheritance mode or spatial and temporal distribution and abundance of haplotypes are capable of influencing diversity estimates and the required sampling effort^67–71^. We had the possibility of partially exploring the temporal variability of the Concepcion population, with the aim of finding previously undetected mitochondrial haplotypes, but it was interesting that although the population of Concepcion was sampled on two occasions, more than 10 years apart (see Suppl. Table S2), lineages A and D were not found. The environmental variability in Concepcion appears to be influenced by seasonal upwelling, the input of fresh water and organic matter from rivers^72,73^, a wide continental shelf and the presence of frontal structures acting as plankton retention and accumulation zone^74^. These environmental factors may exert selective pressures that favors the dominance of the most common lineage within the Intermediate Area (lineage B, Fig. 3). In populations of *Acartia tonsa* that inhabit the northwest Atlantic Ocean, it has been observed that in common garden conditions, the traits of thermal tolerance and body size present seasonal variation that was probably product of a genetic differentiation mediated by drastic seasonality in environmental conditions^75^. Previous studies conducted within the same *A. tonsa* populations had revealed that salinity exerts a significant influence on population genetics, leading to ecological divergence and niche partitioning among mitochondrial lineages^39^. This demonstrates that various environmental conditions in the localities of origin, including seasonal variability, can exert selection pressure on the genetic polymorphism abundance in planktonic organisms, even at reduced geographical scales.

For decades, the presence of *A. tonsa* has been documented along the coast of Chile, with numerous ecological studies conducted on these populations^34,46–49,76^. In these studies, it was expected that *A. tonsa* populations from the Chilean coast would exhibit a range of physiological and life history traits characteristic of cosmopolitan species, such as *A. tonsa* found in other oceans and seas worldwide. These traits include a broad tolerance to environmental fluctuations and the production of large cohorts^27,77,78^. Ecology and physiology studies have shown that populations previously identified as *A. tonsa* off the coast of Chile, including populations studied here, used mechanisms of tolerance and plasticity to face extreme environmental conditions^34,49^, and populations of different biogeographical areas showed signs of adaptation to local conditions of their habitat^34^. When we analyzed the genetic variation of both mitochondrial and nuclear markers between populations and biogeographic areas, we observed high genetic variability within populations, which was consistent with the levels of genetic diversity (Table 1). However, when the genetic variance was partitioned between populations, we observed that only the mitochondrial marker showed a significant effect, whereas the nuclear marker showed a significant differentiation between biogeographic areas. This could be due to the considerable presence of private haplotypes, and that the 18 s marker showed that the Peruvian, Intermediate and Magallanes Provinces did not share haplotypes between them (Fig. 2). On the other hand, although a series of privates’ mitochondrial haplotypes were observed, the most abundant haplotypes were shared and present in more than one biogeographic area, even was possible to find the same haplotype (the most common) in all biogeographic zones (Fig. 2). In general, in the Humboldt current system (SEPO region) it has been observed that species with a prolonged larval phase showed a high genetic diversity associated with a low structuring of their populations, with highly diffused haplotypes capable of tolerating changes in environmental conditions and being present in more than one biogeographic area^16,56^.

The populations studied were composed of some haplotypes and lineages, mainly mitochondrial, capable of being present in different localities, overcoming biogeographic breaks, and their contrasting environmental conditions. However, when we compared the most contrasting populations, we observed that both markers (nuclear and mitochondrial) showed that the localities of Concepcion and Magellan showed the highest significant differences in Фst with respect to the other localities. In this sense, the nucleotide differences between populations were significantly greater with increasing distance between locations (Suppl. Fig. S1), suggesting that isolation by geographic distance played a role in the differentiation between the populations studied, as expected for the Magallanes population (isolated 1200 km from the nearest locality, Hualaihue). It is important to consider that the low number of samples from Magallanes population can influence phylogenetic relationships with other populations and population structure patterns, however a clear pattern of isolation by distance was previously reported for the gastropod *Concholepas concholepas*^79^, which is endemic to the coast of Chile, with a larval phase duration of four months in South-central Chile^80^ and that can reach 12 months in Patagonia^81^. In addition, the presence of significant differences between populations and biogeographic areas, similar to what was found in our study (Table 2 and Suppl. Table S1), suggests that environmental heterogeneity hinders connectivity among populations^79^.

In conclusion, we report the presence of a new species, molecularly recognized for the genus *Acartia* that is endemic to SEPO, specifically from the coasts of Chile, and that so far is morphologically indistinguishable from *Acartia tonsa*. The populations of this new species are more closely related phylogenetically to other endemic species of genus *Acartia* to the Pacific Ocean than to *Acartia tonsa*. Additionally, our results suggest that although connectivity between the populations studied here is possible mainly due to the action of Humboldt current system, the geographical distances and contrasting environmental conditions^34,52^ may be the main structures of the genetic diversity of the copepod populations that inhabit the SEPO region.

## Materials and methods

### Study area and individual collection

In this study, we studied six locations along the Chilean coast covering three biogeographic zones previously proposed in^54^, spanning over 3000 km (Fig. 1), where the presence of the copepod *A. tonsa* has been described. Samples were collected at: (A) Antofagasta (23.6° S 70.4° W) and Tongoy (30.2° S 71.5° W), both situated in the Peruvian Province (4–30° S) which is strongly influenced by year round upwelling and the absence of rivers, (B) off Concepcion (36.5° S 72.9° W) and at the Valdivia estuary (39.8° S 73.2° W), both located in the Intermediate area (transition zone) strongly affected by seasonal upwelling (spring and summer) and high river discharges (autumn and winter). (C) Hualaihue (42° S 72.6° W) and Magallanes (53.1° S 70.9° W), both immersed in the Magellan Province (42–56° S), with a strong influence of a continuous discharge of fresh water along a dismembered coast by fjords and channels with glacial relicts of the last glacial maximum (see details in Fig. 1).

Our research group and other authors have repeatedly characterized the variability of the coastal ocean environment in the localities under study^52,82–84^, accounting for considerable differences between localities in temperature, salinity, PCO_2_, food availability related to the different biogeographical zones. Broadly speaking, higher temperatures prevail in the Peruvian province, which decrease with increasing latitude^84^. Regarding salinity, this is higher in the Peruvian province and decreases in the other provinces, being directly dependent on the contribution of fresh water from the rivers, which increases in winter^82,84^. In the Peruvian province and in the intermediate area, episodic events of high ocean acidification (PCO_2_ ~ 1800 μatm) can be found, mainly associated with upwelling areas and river discharges^52,83^. Finally, it has been observed that the concentration of food is higher and less variable in estuarine localities of the intermediate area than in upwelling areas of the Peruvian province^84^. Detailed information on the main environmental conditions of each population studied in Suppl. Table S2.

Zooplankton samples were collected at each location using gently oblique hauls with a WP2 net (50 cm diameter, 200-µm mesh size), equipped with a non-filtering 1 L cod-end, targeting depths ranging from 10 to 7 m. In the particular case of the Magallanes locality, zooplankton samples were collected from a depth of 50 m to the surface with a filtering cod-end (more details in Suppl. Table S3). The collected samples were preserved in 95% ethanol at − 20 °C. Subsequently, plankton samples were taken to the laboratory for analysis where the identification and sorting of *A. tonsa* females were conducted using taxonomic keys^85^.

### DNA extraction, amplification of molecular markers and genetic analysis

Specimens were selected for genetic analysis, and genomic DNA was extracted from a single individual using the Mollusc DNA Kit (Omega), following the manufacturer's protocols and with the modifications suggested in^20^. Subsequently, the concentration of the extracted DNA was quantified, and the presence of contaminants was verified using a Nanodrop. Finally, the DNA was stored at − 20 °C.

To perform the genetic analyses, two genes were employed: the mitochondrial gene encoding the cytochrome *c* oxidase subunit I (COI) and the nuclear gene encoding the small subunit of the ribosome (18S). The primers used to amplify each gene fragment, along with the observed and expected fragment lengths and the hybridization temperatures during PCR amplification cycle are specified in supplementary materials Table S4. PCR amplification was conducted in a total volume of 30 μL, where included 3 μL of 5X Green GoTaq G2 Flexi Buffer, 3 μL of 25 mM MgCl_2_, 0.5 μL of dNTPs (10 mmol L^−1^), 0.5 μL of each primer (100 pmol µL^−1^), 0.045 units of GoTaq G2 Flexi DNA Polymerase (Promega), and 5 μL of DNA from each individual. The PCR products were verified by electrophoresis on 1.2% agarose gel. Subsequently, the amplified products were sent to Macrogen for sequencing. To avoid ambiguous readings of certain nucleotides, the PCR products were sequenced in both directions (forward and reverse).

### Sequence alignment and phylogenetic analyses

The sequences from both strands of the same individual were aligned to confirm the nucleotide bases and to generate a consensus sequence using the Geneious R11.1.5 (2018). Additionally, species verification was performed using consensus sequences in BLASTn to avoid misidentification of the specimens. The absence of stop codons was confirmed. The final alignments for both gene fragments were conducted using MUSCLE alignment algorithm^86^. The inclusion of *A. tonsa* sequences additional to those generated by us was carried out following a two-step approach: First, we searched for similarity (BLASTn) between our sequences and the sequences available in Genbank and the most similar ones were included. Then (second step), sequences generated in previous research were collected, where the relationship or phylogenetic identity of *A. tonsa* populations had been evaluated. In this case, we included those that were identified as misidentifications and also taxonomic identity corrections^38–45,61,63,64^. This approach allowed us to include the largest number of *A. tonsa* populations from different oceans and seas. In this search, our sequences were the only ones generated from the SEPO region, because the closest ones came from populations on the North Atlantic coasts^44^ or sequences of North Pacific populations misidentified as *A. tonsa*^61^.

Finally, the largest number of sequences of different species of the genus Acartia were included, in order to establish the phylogenetic position of our populations with respect to the species *A. tonsa* within the genus Acartia.

Phylogenetic analyses were performed using maximum likelihood (ML) and Bayesian inference (BI) methods for both analyzed gene fragments. The GTR + G model was selected using Bayesian information criterion (BIC) implemented in Mega11 program^87^ for COI and 18S, respectively. Bayesian analyses were performed using MrBayes V3.2.7^88^. The Markov Chain Monte Carlo chains (MCMC) were run in three simultaneous runs with default heating values, along with pre-established evolutionary models, for 1.0 × 10^6^ generations, sampling tree topologies every 1000 generations for both genes. The first 25% of the trees were discarded as burn-in, and a chain standard deviation of > 0.001 was set to indicate analysis stability. ML inferences were performed following the respective evolutionary model for each gene, using 10,000 bootstraps and 1000 replicates with the IQ-TREE V2.2 web server (http://iqtree.cibiv.univie.ac.at/;^89^). Indices of genetic diversity were calculated for the studied populations, including the number of haplotypes, haplotype diversity, nucleotide diversity, polymorphic sites, and private haplotypes. These calculations were performed using the DnaSp V.6 software^90^. Furthermore, population structuring by location was evaluated using Analysis of Molecular Variance (AMOVA), and pair-wise differentiation between populations (Fst) was determined using Arlequin V.3.5 software^91^) after 10,000 number of iterations. To visualize the haplotypes based on their presence in different locations and the relationships among DNA sequences, a haplotype network was constructed for each molecular marker using the PopArt software^92^. This construction was based on the minimum spanning network criterion^93^ with 1000 bootstrap replicates.

### Species delimitation

Two different analytical methods, the Generalized Mixed Yule Coalescent (GMYC) probability method and the Poisson Tree Process (PTP) model, were used for species delimitation within the *Acartia* genus. In the GMYC analysis, an ultrametric tree of the COI gene was employed, and the BEAST v2.7.4^94^ was used. The following input parameters were selected to represent branching patterns: the GTR substitution model, three categories for gamma-distributed rate variation, local random clock, and Yule model. Four independent runs were conducted, each with 100 million iterations and sampling every 10,000. The convergence of the runs was evaluated using Tracer v1.6^95^. The Maximum Credibility Clade (MCC) tree was determined using Tree Annotator in BEAST v2.7.4^94^ after discarding the first 25% of the trees. The number of delimited species was inferred for each MCC tree using the "gmyc" library^96^ in R^97^. Additionally, species delimitation was performed using the PTP method through the bPTP web server (http://species.h-its.org/ptp/). Bayesian Inference (BI) phylogeny of the COI gene was used as an input for PTP analysis, and 3 × 105 MCMC sampling generations were conducted, with a thinning value of 100 and a burn-in of 25%. The convergence of the MCMC was visually confirmed between the output files.

### Supplementary Information

Supplementary Information.

## Data Availability

DNA sequence data reported in this publication are available through NCBI under accession numbers: mtCOI (OR759115-OR759134), 18 s (OR771437-OR771462).
