# Supplementary material for: Molecular evidence for a new endemic species of Acartia (Copepoda, Calanoida) from the Southeast Pacific coast
Source: Sci Rep. 2024 May 29;14:12366. doi: 10.1038/s41598-024-62080-5 (PMC11137159; doi:10.1038/s41598-024-62080-5)
Supplement: Supplementary file 1 — Supplementary Information. [file 41598_2024_62080_MOESM1_ESM.pdf]

Supplementary materials

Molecular evidence for a new endemic species of *Acartia* (Copepoda, Calanoida) from the Southeast Pacific coast.

Andrés Mesas; Víctor M. Aguilera; Carolina E. González; Ricardo Giesecke; Rubén Escribano; Cristian A. Vargas

Supplementary Tables

Table S1. Mean general pairwise values of differentiation (Φst) between populations. Values above the diagonal correspond to nuclear 18s and under diagonal correspond to mitochondrial COI. Statistically significant comparisons (after Bonferroni correction) and 100,000 iterations are shown in bold.

COI/18s

Locality	Antofagasta	Tongoy	Concepcion	Valdivia	Hualaihue	Magallanes
Antofagasta		**0.27299**	**0.21905**	**0.09107**	**0.34529**	
Tongoy	-0.06660		**0.15219**	0.09278	**0.21413**	
Concepcion	**0.49874**	**0.48394**		0.12066	**0.27556**	
Valdivia	**0.39977**	**0.41852**	0.05458		**0.15680**	
Hualaihue	0.02626	-0.04501	**0.61516**	**0.52739**		
Magallanes	**0.65406**	**0.67874**	**0.48234**	**0.54334**	**0.80898**	

Biogeographic zone	Peruvian	Intermediate	Magellanic
Peruvian		**0.04409**	**0.23344**
Intermediate	**0.42608**		**0.13725**
Magellanic	0.00947	**0.43785**	

Table S2. Main environmental data conditions at the sites where populations studied were sampled. The mean and annual range for each environmental factor are presented. Annual mean ± standard deviation (±S.D.) are presented for Hualaihue and Magallanes.

	Temperature (°C)		Salinity (psu)		Oxygen (mL L-1)		CO_2 (uatm)		Survey water depth (m)
Locality	Mean	Range	Mean	Range	Mean	Range	Mean	Range	
Antofagasta [1]	15.0	14.0-19.0	34.8	34.4-35.0	4.0	1.0-7.0	600.0	360.0-1000.0	15
Tongoy [2]	13.4	12.0-18.0	34.6	34.4-34.7	4.8	0.8-8.0	530.0	360.0-710.0	15
Concepcion [3]	12.0	11.0-14.0	33.8	32.5-34.5	4.0	2.0-6.0	-	-	10
Valdivia [4,5]	11.0	10.5-12.0	31.0	30.0-32.5	5.0	3.0-8.0	-	400.0-1000.0	12
Hualaihue [6]	12.5±1.9	9.3-17.8	30.0 ±0.9	27.0-32.3	6.1±0.9	3.7-8.7	542.8±208.6	171.4-1417.9	5
Magallanes [7]	8.4±0.8	-	30.7±0.3	-	8.9±0.4	-	414.9±33.9	-	Surface

The data of environmental conditions were obtained from published studies and the data for Hualaihue and Magallanes come from measurements that are in progress and whose data are in the process of publication.

1.- Escribano, R., Daneri, G., Farías, L., Gallardo, V. A., González, H. E., Gutiérrez, D., ... & Braun, M. Biological and chemical consequences of the 1997–1998 El Niño in the Chilean coastal upwelling system: a synthesis. DEEP-SEA RES PT II **51**(20-21), 2389-2411 (2004).
2.- Saavedra, L. M., Saldías, G. S., Broitman, B. R., & Vargas, C. A. Carbonate chemistry dynamics in shellfish farming areas along the Chilean coast: natural ranges and biological implications. ICES J. Mar. Sci. **78**(1), 323-339 (2021).
3.- Sobarzo, M., Bravo, L., Donoso, D., Garcés-Vargas, J., & Schneider, W. Coastal upwelling and seasonal cycles that influence the water column over the continental shelf off central Chile. Prog. Oceanogr. **75**(3), 363-382 (2007).
4.- Garcés-Vargas, J., Ruiz, M., Pardo, L. M., Nuñez, S., & Pérez-Santos, I. Hydrographic features of Valdivia river estuary south-central Chile. Lat. Am. J. Aquat. Res. **41**(1), 113-125 (2013).
5.- Osma, N., Latorre-Melín, L., Jacob, B., Contreras, P. Y., von Dassow, P., & Vargas, C. A. Response of phytoplankton assemblages from naturally acidic coastal ecosystems to elevated pCO2. Front. Mar. sci. **7**, 323 (2020).
6.- Vargas et al. Unpublished data generated from Coastal environmental monitoring, Coastal Social-Ecological Millennium Institute (SECOS), Universidad de Concepción & P. Universidad Católica de Chile, Chile.
7.- Giesecke et al. Unpublished data generated from EQM190013 ANID project, Environmental monitoring of fjords and canals of Magallanes region, Universidad Austral de Chile, Chile. https://www.starm.cl/

Table S3. Methodological details of sampling in the studied populations.

Locality	Sampling season	Sampling year	Technique of sampling	Mesh size
Antofagasta	Spring	2015	WP2 with oblique haul	200 µm
Tongoy	Spring	2015	WP2 with oblique haul	200 µm
Concepcion	Spring	2012 and 2023	WP2 with oblique haul	200 µm
Valdivia	Spring	2012	WP2 with oblique haul	200 µm
Hualaihue	Spring	2022	WP2 with oblique haul	200 µm
Magallanes	Spring	2022	WP2 with oblique haul	200 µm

Table S4. Primer details and thermocycler PCR protocols for mitochondrial and nuclear markers

Mitochondrial marker
COI
700bp
Forward LCO1490: 5'-GGTCAACAAATCATAAAGATATTGG-3'
Reverse HCO2198: 5'-TAAACTTCAGGGTGACCAAAAAATCA-3'
Folmer et al. 1994

Thermocycler PCR protocol	Temperature(°C)	Time (s)	
Initial denaturation	94	60	
Denaturation	94	40	
Annealing	48	40	32 cycles
Extention	72	40	
Final extention	72	60	
Final hold	4	∞	

Nuclear marker
18s
1800bp
Forward 18A1mod: 5'-CTGGTTGATCCTGCCAGTCATATGC-3'
Reverse 1800mod: 5'-GATCCTTCCGCAGGTTCACCTACG-3'
Raupach et al. 2009

Thermocycler PCR protocol	Temperature(°C)	Time (s)	
Initial denaturation	94	60	
Denaturation	94	40	
Annealing	54	60	37 cycles
Extention	72	60	
Final extention	72	90	
Final hold	4	∞	

Supplementary Figures

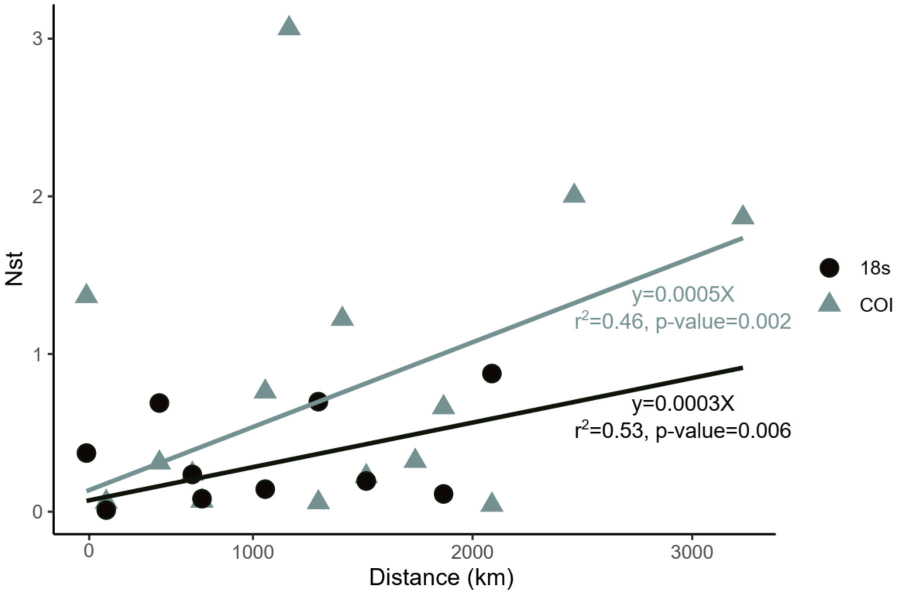

Fig. S1. Relationship between linearized genetic differentiation (Nst) and geographic distances among the populations studied along the Peruvian province, Intermediate area and the Magellanic province.

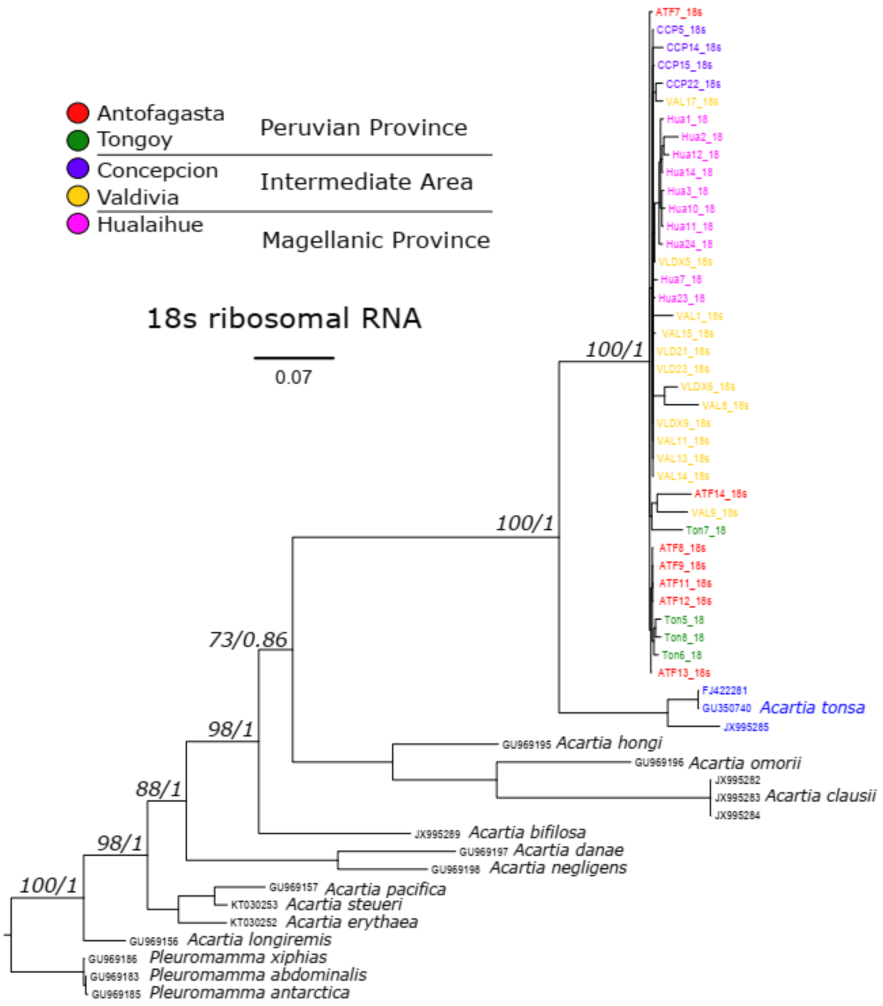

Fig. S2. Bayesian phylogeny performed with 58 sequences of species of genus *Acartia sp.*, including 38 sequences obtained from the populations under study (Antofasta, Tongoy, Concepcion, Valdivia, and Hualaihue). Maximum likelihood / Bayesian posterior probability support values were labeled in each node.
